# Fear of cancer recurrence in long-term colorectal cancer survivors: a nationwide cross-sectional study

**DOI:** 10.1007/s11764-025-01746-z

**Published:** 2025-01-27

**Authors:** Johanne Dam Lyhne, Lars Henrik Jensen, Per Fink, Signe Timm, Lisbeth Frostholm, Allan ‘Ben’ Smith

**Affiliations:** 1 Department of Oncology, Lillebaelt Hospital – Vejle, University Hospital of Southern Denmark, Beriderbakken 4, 7100 Vejle, Denmark; 2 Department for Functional Disorders, Aarhus University Hospital, Palle Juul-Jensens, Boulevard 11, Indgang A, Plan 9, A903, 8200 Aarhus N, Denmark; 3 Department of Clinical Medicine, Aarhus University, Palle Juul-Jensens Boulevard 11, Hospital Building A, 10th floor, 8200 Aarhus N, Denmark; 4 The Daffodil Centre, The University of Sydney, A Joint Venture With Cancer Council NSW, 153 Dowling St, Woolloomooloo, Sydney, NSW 2011 Australia

**Keywords:** Patient-reported outcome measures, Health anxiety, Survivorship, Late effects, Odds ratio, Colorectal cancer

## Abstract

**Purpose:**

Knowledge about fear of cancer recurrence (FCR) among recurrence-free long-term colorectal cancer survivors (CRCS) is limited. This national cross-sectional study aimed to (1) assess the prevalence and correlates of FCR among CRCS; (2) investigate associations between colorectal cancer-specific symptoms and FCR; and (3) identify predictors of interest in engaging in FCR treatment.

**Methods:**

We identified 9638 living Danish CRCS, age above 18 years, diagnosed between 2014 and 2018 through the Danish Clinical Registries. Electronic surveys were distributed between May 2023 and May 2024. FCR was measured on the Fear of Cancer Recurrence Inventory – Short Form (FCRI-SF). Associations with colorectal cancer-specific physical symptoms and psychological symptoms were analyzed using logistic regression models.

**Results:**

Of 5480 respondents (56.9%; mean age: 73, range (30–99; 42% female), 5.3% of survivors reported clinical FCR (cFCR). In multivariate analyses, having severe abdominal pain (OR 8.7 (95% CI 4.8–15.8)), abdominal bloating, tension, or heaviness (OR 10.0 (95% CI (6.1–16.3)) and tiredness (OR 7.1 (95% CI (4.1–12.1)) were associated with increased odds of cFCR, as were psychological symptoms (health anxiety; OR 19.7 (95% CI (13.5–28.6)), anxiety; OR 11.2 (95% CI (6.4–19.6)), depression; OR 5.5 (95% CI (2.6–11.9)) compared to no FCR. Among those with cFCR, 75% were interested in treatment, with higher interest among males and chemotherapy recipients.

**Conclusion:**

FCR severity is strongly associated with specific colorectal symptoms, tiredness, and psychological symptoms.

**Implications for Cancer Survivors:**

Addressing cancer-specific physical symptoms may be a promising strategy for reducing FCR.

**Supplementary Information:**

The online version contains supplementary material available at 10.1007/s11764-025-01746-z.

## Background

Advancements in anticancer treatments have led to a growing population of colorectal cancer survivors (CRCS) and an extended survivorship phase. Although long-term survivors (> 5 years post-diagnosis) are no longer burdened by active treatment and routine follow-up appointments, they continue to face the uncertainty of potential cancer recurrence. The fear of recurrence (FCR), defined as “fear, worry or concern that cancer will come back or progress” [1] remains a significant health-related concern for most cancer survivors, regardless of cancer type or trajectory [2]. Addressing FCR is thus recognized as one of the pressing unmet supportive needs in cancer care [3, 4].

A review [2] of mixed cancer survivors found no variation in FCR by time since diagnosis, suggesting that, in the absence of intervention, FCR tends to persist over time, negatively impacting quality of life [5]. Among 676 colorectal cancer patients, the review reported a prevalence of clinical FCR of 16% using the Fear of Cancer Recurrence Inventory-Short Form (FCRI-SF) [6], and similar prevalence has been observed in other studies of long-term CRCS [7, 8].

Physical symptoms might act as persistent reminders of the disease or as potential indicators of cancer recurrence, leading to elevated FCR, emotional distress [9], and decreased quality of life [10], especially when associated with hyper-vigilance toward bodily symptoms [11]. Distinguishing physical symptoms that are more likely to trigger FCR or be triggered by FCR has not been thoroughly investigated in CRCS. We hypothesized that colorectal cancer-specific symptoms would have a stronger association with FCR compared to symptoms not specifically related to colorectal cancer.

While the FCRI-SF and similar screening tools provide useful estimates of the prevalence and severity of FCR, they do not capture its impact on daily life or assess survivors’ interest in engaging in FCR treatment [12]. It remains unclear whether the characteristics of survivors with clinical FCR interested in FCR treatment differ from those with clinical FCR who are unlikely to accept treatment. We hypothesized that specific characteristics like female gender and time to next follow-up may predict survivors’ interest in FCR treatment.

This cross-sectional study contributes to the existing knowledge of colorectal cancer survivorship by providing nationwide, cross-sectional data on prevalence and associations of physical and psychological late effects in recurrence-free, long-term CRCS, with a focus on FCR. The primary objectives of this study were to evaluate, in a population-based cohort of CRCS approximately 5–10 years post-diagnosis: (1) the prevalence and correlates of various levels of FCR, (2) the association between colorectal cancer-specific symptoms and FCR, (3) the association between psychological symptoms and FCR, and (4) predictors of survivors with clinical FCR interested in engaging in FCR treatment.

## Method

### Study design

The study adopted a population-based, cross-sectional design and followed the COSMIN Study Design checklist for Patient-Reported Outcome Measures (PROMs) and STROBE guidelines for cross-sectional studies [13].

### Study population

The target population comprised all Danish CRCS aged 18 or above, diagnosed between March 2014 and December 2018, treated surgically with curative intent according to the Danish Colorectal Cancer Group’s (DCCG) prospective database, which holds information on 99% of patients with colorectal cancer in Denmark [14]. The study was approved by The Regional Committees on Health Research Ethics for Southern Denmark (Project-ID: S-20190061).

### Study procedure

Electronic surveys from “Vejle Hospital” were distributed to a digital citizen mailbox between May 2023 and May 2024. In case of no response, two reminders were sent out after 10 and 20 days. Reasons for non-participation were occasionally reported by phone or email to the primary investigator. Death was noted by automatic email return. Consent for the use of responses in research was obtained through the survey. The completed questionnaires were directly uploaded into the REDCap database for clinical research.

### The survey

Participants were invited to complete an electronic questionnaire to screen for physical and psychological symptoms and health-related quality of life (HRQoL).

### Patient-Reported Outcomes (PROMs)

1. Fear of cancer recurrence

FCR was measured by the 9-item Fear of Cancer Recurrence Inventory-Short Form (FCRI-SF) [6, 15]. The FCRI-SF is the most widely validated and used screening tool with response categories ranging from not at all/never (0) to a great deal/all the time (4). Scale scores range from 0 to 36 with higher scores indicating greater FCR severity. Originally, two cutoff scores were proposed and validated (a cutoff of 13/36 to indicate any level of FCR and a cutoff of 16/36 to indicate potential severe FCR). More recently, a cutoff of 22 has been suggested for identifying clinical cases of FCR according to clinical interview [16]. The sensitivity of this high cutoff has been reported to be 90% and the specificity 83.3%. Survivors were categorized into groups according to FCRI-SF score: nonclinical FCR (nFCR, FCRI-SF score ≤ 13), subclinical FCR (sFCR, FCRI-SF score 13–21), and clinical FCR (cFCR, FCRI-SF score ≥ 22). All respondents were informed about their level of FCR based on the FCRI-SF.

2. Anxiety and depression

Anxiety and depression were measured with the Symptom Checklist-90-R (SCL) [17] subscales [18] for anxiety (SCL-anx, 4 items) and depression (SCL-dep, 6 items). The SCL measures symptoms of anxiety and depression during the previous 4 weeks on a scale ranging from 0 (not at all) to 4 (extremely). Scale scores range from 0 to 16 for anxiety and 0 to 24 for depression. Higher scores indicate higher severity of symptoms. The SCL has undergone psychometric testing as a part of the Common Mental Disorder Questionnaire (CMDQ) in a primary care setting with 701 patients [18, 19]. SCLs ability to correctly classify those with ICD-10 diagnoses above/below cutoff compared to psychiatric research interview (SCAN) were excellent with an area under the curve for SCL-dep (cutoff ≥ 9) on 0.88 (95% CI 0.84–0.91) and for SCL-anx (cutoff ≥ 6) on 0.87 (95% CI 0.82–0.92) [18].

3. Health anxiety

Health anxiety was measured with the Whiteley-6-R Index [20]. The Whiteley-6-R Index measures symptom of health anxiety during the previous 4 weeks on a scale ranging from 0 (not at all) to 4 (extremely). Scale scores range from 0 to 24. Higher scores indicate higher severity of symptoms. The Whiteley-6-R has been validated in a general population sample of 9656 individuals showing good content validity (relevance) with a low number of missing responses on all items, good construct validity evaluated by structural validity (exploratory factor analysis and confirmatory factor analysis), and good convergent and discriminant validity (hypothesis testing) [20]. Criterion validity has been examined with excellent results against an interview-based diagnosis of health anxiety as a gold standard in a sample of 1590 individuals in the general population with an area under the curve on 0.88 (95% CI 0.84–0.92) [20]. No established cutoff for the Whiteley-6-R has been published to date. However, a recent randomized controlled trial from the Department of Functional Disorders at Aarhus University Hospital, Denmark [21], used a cutoff of > 21.4 (scale 0–100) on the Whiteley-7 Index, which corresponds to ≥ 6 on the Whiteley-6-R (scale 0–24). This cutoff is adopted in the present study and will be assessed further in an upcoming publication.

4. Physical symptoms

Physical symptoms were identified by the Bodily Distress Syndrome (BDS) checklist [22]. The BDS checklist measures physical load on a scale from 0 (not at all) to 4 (extremely). The 25-item checklist covers symptoms related to cardiopulmonary symptoms, bowel symptoms, musculoskeletal symptoms, and general symptoms, including tiredness.

Colorectal cancer-specific symptoms were defined using four items from the BDS checklist: frequent, loose stools; abdominal pain; abdominal bloating, tension, or heaviness, and diarrhea. These were supplemented with three additional items covering well-known late effects after colorectal cancer with the same response categories: Involuntary passage of air and/or loose stool, urgent bowel movement, and difficulty emptying the bowels during toilet visits. The remaining items from the BDS checklist were categorized as non-colorectal cancer-specific symptoms and were supplemented with four new items: altered sexual function, involuntary bladder leakage, frequent urination, and difficulty emptying the bladder as sexual and urinary symptoms are also common after colorectal cancer, but not assessed by the BDS checklist.

5. Health state and health-related quality of life

Health state was assessed by the EQ-5D-5L [23], which includes five domains (mobility, self-care, usual activities, pain, and anxiety/depression). Each domain is assessed on a five-level scale, ranging from 1 (no problems) to 5 (extreme problems). HRQoL was assessed using the single visual analogue scale (EQ-VAS) where participants were asked to self-rate their overall quality of life TODAY on a VAS scale ranging from 0 to 100 with 0 being “The worst health you can imagine” and 100 being “The best health you can imagine.” A difference 10 out of 100 points between groups was considered significant.

6. Clinical characteristics and sociodemographic information

Purpose-designed questions regarding the time since the last cancer surveillance visit, time until the next visit, and the receipt of chemotherapy and/or radiation were included. Furthermore, the participants were asked to report marital status, employment status, educational level, citizenship (Danish or other), and children (yes or no). In case of a FCRI-SF score ≥ 22, participants were automatically asked whether they were interested in information about a trial specifically developed for treatment of FCR [24].

### Registry data

The following demographic and clinical characteristics were available through the Danish Clinical Quality Program – National Clinical Registries (RKKP): age and gender, cancer type (colon or rectum), date of diagnosis, tumor stage, and if the patient was diagnosed as consequence of colorectal cancer screening (yes/no).

### Data cleaning

Responses were excluded in case of cancer recurrence, or if “dementia,” “terminal/too somatic ill,” or “dead” were communicated by caregivers to the primary investigator by telephone or email. If the survivor indicated “no memory of cancer” (often surgical removal of a polyp) or “do not want to participate” (often because the participant did not want to be reminded of the cancer period) their response was excluded. Responses without consent for research use were also excluded as were cases with incomplete demographic data. Finally, completion of all FCRI-SF items was mandatory; however, one respondent exited the questionnaire before completing the FCRI-SF and was subsequently excluded from the analysis.

### Statistical analyses

Descriptive statistics were reported for each FCR severity group as absolute number/percentages or range for demographic measures. To minimize the risk of type 1 errors, differences in percentages between groups exceeding 10% were deemed clinically relevant rather than relying on statistical testing. Factors predicting interest in engaging in treatment for FCR were investigated with chi-squared test and one-way ANOVA. Logistic regression models were used to estimate the odds ratio (OR) of having sFCR and cFCR compared to nFCR depending on symptom severity, adjusted for possible confounders (gender, age, marital status, education, employment status, having children, language, and citizenship). A Directed Acyclic Graph (DAG) [25] was made at DAGitty.net to identify the confounders to include in the minimal adjustment set (Supplementary File 1, Fig. 1). The logistic regression models were extended to include symptoms that were weakly correlated (Pearson correlation coefficient < 0.3) with each other to avoid multi-collinearity. Sensitivity analyses excluding patients with survival durations under 5 years were conducted for all outcomes. Symptoms of anxiety and depression were dichotomized according to established cutoffs and included in the models. All physical symptoms were divided into severity with “No symptoms” corresponding to 0, “Mild symptoms” corresponding to 1 or 2, and “Severe symptoms” corresponding to 3 or 4. In case of missing items within the PROMs, the item was replaced with a 0 indicating a conservative approach assuming that the symptom in question was not present. If more than half of items were missing, the sum score was not calculated. No imputation was performed. Statistical analyses were performed using STATA 17 (Stata Corp., College Station, TX, USA).

**Fig. 1:**
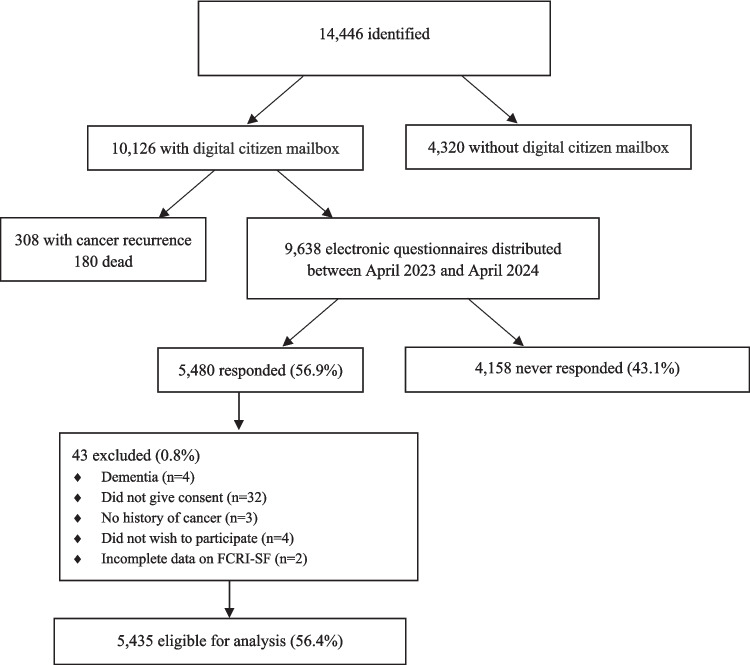
Participant flowchart

## Results

A total of 14,446 CRCs were identified, of whom 10,126 had an electronic mailbox. During the survey period from May 2023 to May 2024, 180 CRCS were reported deceased, and 308 CRCS reported a cancer recurrence. Of the remaining 9638 questionnaires, 5480 were responded (56.4%). Of these, 45 (0.8%) were excluded due to dementia (*n* = 4), no recall of cancer (*n* = 3), incomplete FCRI-SF data (*n* = 2), no wish to participate (*n* = 4), or no consent to participate (*n* = 32). While we aimed to recruit patients 5–10 years post-diagnosis, 226 (4.2%) were between 4.4 and 5 years post-diagnosis. Flowchart is presented in Fig. 1.

Demographic and clinical characteristics, stratified by FCR severity category, are listed in Table 1. The mean FCRI-SF score across the cohort was 9.8 (SD = 6.6). The prevalence of cases with sFCR was 28.6% and the prevalence of cFCR was 5.3%. Patients with cFCR were more likely to be female, employed, and not participating in colorectal cancer screening programs. They were also characterized by longer intervals since or until their next cancer follow-up, prior chemotherapy treatment, poorer health state—particularly in dimensions of pain and anxiety—and reduced HRQoL. No clinically relevant differences were observed between groups regarding cancer type, time since diagnosis, radiotherapy, disease stage (metastatic or not), citizenship, or parental status. There was a trend suggesting that patients with cFCR were younger, unpartnered, and less likely to hold a higher educational degree. Sensitivity analyses excluding patients with survival durations under 5 years did not alter the results (data not shown).

**Table 1:** Demographic and clinical characteristics for responders divided according to severity level of FCR

Characteristics	No FCR (FCRI-SF < 13) *n* = 3592	Subclinical FCR (FCRI-SF 13–21) *n* = 1555	Clinical FCR (FCRI-SF ≥ 22) *n* = 288	Total population *n* = 5435
Registry data				
Age in years, mean (sd), range (min–max)	74 (8.3), 30–99	70 (9.2), 31–94	67 (11.1), 34–98	73 (9.1), 30–99
Female gender	1340 (37.3)	786 (50.5)	164 (57.0)	2291 (42.1)
Cancer type				
Colon	2388 (66.5)	1034 (66.5)	201 (69.8)	3624 (66.7)
Rectum	1204 (33.5)	521 (33.5)	87 (30.2)	1813 (33.4)
Years since diagnosis, mean (sd), range (min–max)	7.3 (1.4), 4.4–10.1	7.1 (1.4), 4.4–10.1	7.0 (1.5), 4.4–9.9	7.2 (1.4), 4.4–10.1
Metastatic disease at time of diagnosis				
No	3394 (94.5)	1441 (92.7)	268 (93.1)	5105 (93.9)
Yes	166 (4.6)	93 (6.0)	19 (6.6)	278 (5.1)
Missing	32 (0.9)	21 (1.4)	1 (0.4)	54 (1.0)
Participated in colorectal cancer screening	1465 (40.8)	572 (36.8)	86 (29.9)	2123 (39.1)
Self-reported data				
Time since last follow-up				
> 1 year	2618 (72.9)	1096 (70.5)	177 (61.5)	3892 (71.6)
3–12 months	724 (20.2)	345 (22.2)	77 (26.7)	1146 (21.1)
1 week–3 months	225 (6.3)	103 (6.6)	33 (11.5)	361 (6.6)
< 1 week	25 (0.7)	11 (0.7)	1 (0.4)	37 (0.7)
Time to next follow-up				
< 1 week	19 (0.5)	15 (1.0)	4 (1.4)	38 (0.7)
1 weeks–3 mo	171 (4.8)	84 (5.4)	20 (6.9)	275 (5.1)
3 mo.–12 mo	279 (7.8)	177 (11.4)	35 (12.2)	491 (9.0)
> 1 year	862 (24.0)	465 (29.9)	102 (35.4)	1429 (26.3)
Follow-up ended	2261 (63.0)	814 (52.4)	127 (44.1)	3203 (58.9)
Chemotherapy received	1252 (34.9)	696 (44.8)	141 (49.0)	2089 (38.4)
Radiotherapy received	233 (6.5)	103 (6.6)	21 (7.3)	357 (6.6)
Health state (best state)*				
Mobility	2767 (77.0)	1115 (71.7)	170 (59.0)	4052 (74.5)
Self-care	3395 (94.5)	1431 (92.0)	233 (80.9)	5059 (93.1)
Activity	2738 (76.2)	987 (63.5)	138 (47.9)	3863 (71.1)
Pain	2507 (69.8)	733 (47.1)	91 (31.6)	3331 (61.3)
Anxiety	3229 (89.9)	964 (62.0)	86 (29.9)	4279 (78.7)
HrQoL, mean (sd)	79.7 (18.2)	72.7 (18.9)	62.1 (23.0)	76.8 (19.2)
Marital status				
Married	2464 (68.6)	983 (63.2)	172 (59.7)	3619 (66.8)
Not married	200 (5.6)	135 (8.7)	28 (9.7)	363 (6.7)
Divorced/separated	244 (6.8)	144 (9.3)	43 (14.9)	431 (8.0)
Widowed	484 (13.5)	175 (11.3)	27 (9.4)	686 (12.7)
Living together	191 (5.3)	112 (7.2)	18 (6.3)	321 (5.9)
Missing	9 (0.3)	6 (0.4)	0 (0.0)	15 (0.3)
Employment status				
Employed	655 (18.2)	434 (27.9)	107 (37.2)	1196 (22.0)
Has been employed	2834 (78.9)	1083 (69.7)	174 (60.4)	4091 (75.2)
Never been employed	44 (1.2)	15 (1.0)	3 (1.0)	62 (1.1)
Missing	59 (1.6)	23 (1.5)	4 (1.4)	86 (1.6)
Level of education				
Low (0–3 years)	616 (17.2)	256 (16.5)	58 (20.1)	930 (17.1)
Middle (3–5 years)	1555 (43.3)	676 (43.5)	127 (44.1)	2358 (43.4)
High (5 years or above)	1205 (33.6)	530 (34.1)	84 (29.2)	1819 (33.5)
Other education	187 (5.2)	72 (4.6)	15 (5.2)	274 (5.0)
Missing	29 (0.81)	21 (1.4)	4 (1.4)	54 (1.0)
Citizenship				
Danish	3461 (96.4)	1496 (96.2)	275 (95.5)	5232 (96.3)
Not Danish	115 (3.2)	50 (3.2)	11 (3.8)	176 (3.2)
Missing	16 (0.5)	9 (0.6)	2 (0.7)	37 (0.7)
Children	3197 (89.0)	1405 (90.4)	264 (91.7)	4866 (89.5)
Missing	30 (0.8)	10 (0.6)	3 (1.0)	43 (0.8)

*Only best state (1 out of 5 EQ-5D-5L states) is shown. Exact numbers on remaining states can be found in Supplementary File 2, Table 2a

Percentages refer to distribution of states within each domain

Data are presented as *n* (%) unless otherwise stated

Mo., months; *HrQoL*, health-related quality of life

### Correlates of FCR category

**Fig. 2:**
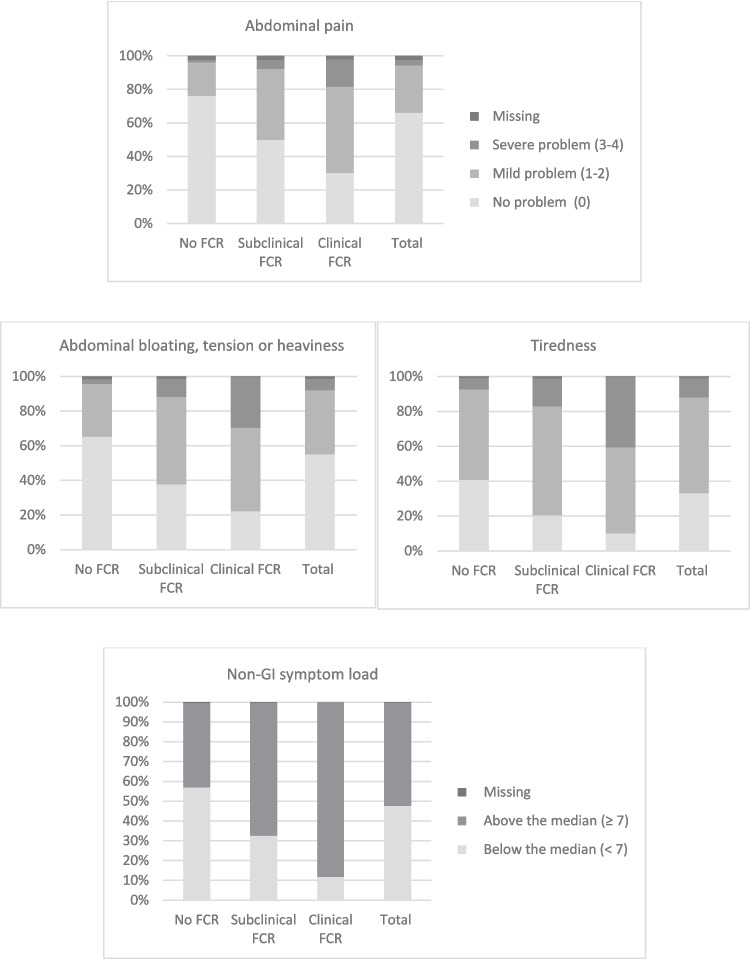
Cases of colorectal cancer-specific symptoms, tiredness, and non-gastrointestinal symptom load according to fear of cancer recurrence level

FCR category was significantly (*p* < 0.01) associated to all reported physical and psychological symptoms (see Fig. 2). Exact numbers are presented in Supplementary File 2, Tables 2b, and 2c. Missing data were equally distributed between groups.

**Table 2:** Adjusted odds ratio of subclinical (sFCR, FCRI-SF 13–22) and clinical (cFCR, FCRI-SF ≥ 22) fear of cancer recurrence and presence of psychological symptoms above cutoff (reference group: nFCR, FCRI-SF ≤ 13)

Psychological symptoms	Adjusted* odds ratio (95% CI) for reporting sFCR and cFCR, respectively		
	No symptoms	sFCR	cFCR
Health anxiety	Reference	3.7 (3.0–4.6)	19.7 (13.5–28.6)
Anxiety	Reference	2.7 (1.8–4.2)	11.2 (6.4–19.6)
Depression	Reference	2.4 (1.4–4.0)	5.5 (2.6–11.9)

*According to the DAG model (see Supplementary File 1) we adjusted for age, gender, treatment type (chemotherapy, radiation), demographics (employment status, marital status, children, and education) and clinical characteristics (diagnosed through screening, ongoing cancer follow-up, and time since diagnosis) and variables (physical and psychological) with a correlation ≤ 0.3

**Table 3:** Adjusted odds ratio for reporting subclinical (sFCR, FCRI-SF 13–22) and clinical (cFCR, FCRI-SF ≥ 22) fear of cancer recurrence with mild or severe colorectal cancer-specific symptoms (reference group: nFCR, FCRI-SF ≤ 13)

Colorectal cancer-specific symptoms	Adjusted* odds ratio (95% CI) for reporting sFCR and cFCR, respectively				
		sFCR (FCR 13–22)		cFCR (≥ 22)	
	No problem	Mild problem	Severe problem	Mild problem	Severe problem
Frequent, loose stools	Reference	1.6 (1.3–1.8)	1.9 (1.5–2.3)	1.6 (1.1–2.4)	3.3 (2.0–5.3)
Abdominal pain	Reference	2.4 (2.0–2.8)	2.8 (1.9–4.3)	3.6 (2.6–5.1)	8.7 (4.8–15.8)
Abdominal bloating, tension, or heaviness	Reference	2.1 (1.8–2.5)	3.0 (2.2–4.1)	2.7 (1.9–3.9)	10.0 (6.1–16.3)
Diarrhea	Reference	1.4 (1.2–1.6)	1.3 (1.0–1.8)	1.7 (1.2–2.5)	3.7 (2.2–6.5)
Involuntary passage of gas/loose stool	Reference	1.5 (1.3–1.7)	2.0 (1.5–2.6)	1.4 (1.0–2.1)	2.2 (1.3–3.7)
Difficulty emptying bowels during toilet visits	Reference	1.4 (1.2–1.7)	2.0 (1.5–2.6)	1.2 (0.8–1.8)	2.2 (1.3–3.7)
Defecation urgency	Reference	1.5 (1.3–1.7)	2.1 (1.7–2.7)	1.2 (0.8–1.8)	2.5 (1.5–4.0)
Non-colorectal cancer-specific symptoms	Adjusted* odds ratio (95% CI) for reporting sFCR and cFCR, respectively				
		sFCR (FCR 13–22)		cFCR (≥ 22)	
	No problem	Mild problem	Severe problem	Mild problem	Severe problem
Involuntary urination	Reference	1.0 (0.8–1.1)	0.8 (0.5–1.2)	0.8 (0.5–1.3)	1.1 (0.5–2.2)
Frequent urination	Reference	1.1 (0.9–1.3)	1.0 (0.7–1.3)	0.7 (0.5–1.1)	1.0 (0.6–1.8)
Difficulty emptying bladder	Reference	1.1 (0.9–1.3)	0.6 (0.4–0.8)	0.7 (0.5–1.1)	0.7 (0.4–1.4)
Sexual dysfunction	Reference	1.1 (0.9–1.4)	1.2 (1.0–1.6)	0.9 (0.5–1.4)	1.2 (0.7–2.0)
Neuropathy	Reference	1.2 (1.0–1.5)	1.1 (0.8–1.6)	1.1 (0.8–1.7)	0.9 (0.5–1.7)
Tiredness	Reference	2.0 (1.7–2.4)	2.3 (1.8–3.0)	2.6 (1.6–4.1)	7.1 (4.1–12.1)
Non-GI symptom load^	Reference	2.0 (1.7–2.3)		4.7 (3.0–7.2)	

*According to the DAG model (see Supplementary File 1) we adjusted for age, gender, treatment type (chemotherapy, radiation), demographics (employment status, marital status, children, and education) and clinical characteristics (diagnosed through screening, ongoing cancer follow-up, and time since diagnosis) and variables (physical and psychological) with a correlation ≤ 0.3

^Non-GI symptom load is assessed as physical symptoms on the BDS checklist aside from GI-symptoms (cardiopulmonary symptoms, musculoskeletal symptoms and general symptoms) with a cutoff defined as above or below the median.

For ORs where the 95% CI includes 1, the association is non-significant

### Correlates of FCR category with psychological symptoms

In the overall sample, the prevalence of cases with health anxiety (scores above a cutoff ≥ 6) was 16.0%, the prevalence of cases with anxiety (scores above a cutoff of ≥ 6) was 4.5% and the prevalence of cases with depression (scores above cutoff of ≥ 9) was 2.9%. Patients with health anxiety score above cutoff were more likely to be in the cFCR group (aOR 19.7 (95% CI (13.5–28.6)) as were patients with anxiety scores above cutoff (aOR 11.2 (95% CI (6.4–19.6)) and patients with depression scores above cutoff (aOR 5.5 (95% CI (2.6–11.9)). For severe suicidal risk, the odds of being in the cFCR group was also elevated (aOR 11.0 (95% CI 1.8–66.4)). The adjusted odds ratio for sFCR was markedly lower for each psychological symptom (see Table 2).

### Correlates of FCR category with physical symptoms

Four colorectal cancer-specific symptoms (Loose stool, involuntary passing of gas and/or stools, difficulty emptying the bowel during toilet visits, and defecation urgency) were reported as severe by approximately 1/10 of participants. Severe sexual dysfunction was experienced by 17.6%. Severe tiredness was reported by 11.2%. Patients having severe abdominal bloating, tension, or heaviness was ten times more likely to be in the cFCR group compared to the nFCR group (aOR 10.0, 95% CI (6.1–16.3) while patients with severe abdominal pain had a ninefold increase of cFCR (aOR 8.7, 95% CI(4.8–15.8). Patients with severe tiredness was 7 times more likely to be in the cFCR group compared to the nFCR group (aOR 7.1, 95% CI (4.1–12.1)). The same symptoms were associated with at least a twofold risk of being in the sFCR group relative to nFCR.

Lower odds ratios were found between other symptoms of bowel dysfunction, whereas non-colorectal cancer-specific symptoms like urinary dysfunction, sexual dysfunction, and neuropathy were not associated with an increased odds ratio of being in the cFCR group (Table 3). Reporting symptom burden above the median, measured by non-gastrointestinal symptoms on the BDS checklist, showed an aOR of 4.7 (95% CI (3.0–7.2)) of being in the cFCR group.

For ORs where the 95% CI includes 1, the association is non-significant.

Of the 288 CRCS with cFCR, 215 (74.7%) were interested in engaging in treatment. Compared to those not interested, the cohort interested in FCR treatment was characterized by a higher proportion of males, employed CRCS, chemotherapy recipients, CRCS closer to their last follow-up, and those reporting a poorer overall health state (Table 4). Interest in engaging in FCR treatment was not associated with any physical and psychological symptoms (Supplementary File 3). Results remained consistent after excluding patients with survival durations under five years (data not shown).

**Table 4:** Characteristics of colorectal cancer survivors with clinical FCR interested in engaging in treatment

	Interested in engaging in treatment for FCR *n* = 215	Not interested in engaging in treatment for FCR *n* = 73
Age, mean (sd), range (min–max)	66.1 (11.5), 34–98	69.0 (9.4), 46–93
Female gender	115 (53.5)	49 (67.1)
Time since the last follow-up		
> 1 year	124 (57.7)	53 (72.6)
3–12 months	62 (28.8)	15 (20.6)
1 week–3 months	28 (13)	5 (6.9)
< 1 week	1 (0.5)	0
Time to next follow-up		
< 1 week	3 (1.4)	1 (1.4)
1 weeks–3 months	16 (7.4)	4 (5.5)
3 months–12 months	26 (12.1)	9 (12.3)
> 1 year	79 (36.7)	23 (31.5)
Follow-up has ended	91 (42.3)	36 (49.3)
Chemotherapy received	113 (52.6)	28 (38.4)
Health state (best state)*		
Mobility	119 (55.4%)	51 (69.9%)
Self-care	171 (79.5%)	62 (84.9%)
Activity	93 (43.3%)	45 (61.6%)
Pain	61 (28.4%)	30 (41.1%)
Anxiety	61 (38.4%)	25 (34.3%)
HrQoL, mean (sd)	61 (22.8)	65 (23.6)
Employment status		
Employed	86 (40.0)	21 (28.8)
Has been employed	123 (57.2)	51 (69.9)
Has never been employed	2 (0.9)	1 (1.4)
Missing	4 (1.9)	0 (0.0)
Level of education		
Low (0–3 years)	40 (18.6)	18 (24.7)
Middle (3–5 years)	95 (44.2)	32 (43.8)
High (5 years or above)	66 (30.7)	18 (24.7)
Other education	10 (4.7)	5 (6.9)
Missing	4 (1.9)	0
Participated in colorectal cancer screening	62 (28.8)	24 (32.9)
Baseline FCRI-SF, mean (sd)	25.1 (2.8)	24.0 (2.4)

Data are presented as *n* (%) unless otherwise stated

*HrQoL*, health-related quality of life

## Discussion

This population-based cross-sectional study reveals that FCR remains a prevalent and multifaceted concern among long-term CRCS. Our findings confirm previously reported associations of FCR with clinical and demographic factors like younger age [26], female gender [27], receiving chemotherapy [8], and not participating in screening programs as shown for young women with early-stage breast cancer [28]. They also confirm the lack of association between FCR and cancer type [29] and years since diagnosis [2].

Findings also highlight that patients with specific colorectal cancer symptoms (severe abdominal pain and severe abdominal bloating, tension, or heaviness), severe tiredness, and psychological symptoms (health anxiety, anxiety and depression) are markedly more likely to be in the cFCR group relative the nFCR group.

While the relationship between anxiety and depression and FCR is well established [5, 7, 30], the association between health anxiety and FCR remains underexplored. The significant overlap between patients reporting FCR and patients reporting health anxiety suggests shared underlying constructs. Further research is required to delineate their similarities and unique features to ensure the validity of existing PROMs measuring health anxiety and FCR, and the effectiveness of current treatment interventions.

Concerning the association between specific colorectal cancer symptoms and FCR, non-specified side effects have been associated with FCR in a smaller population of 134 CRCS [31] and the association between physical symptoms in general and FCR has been reported in other diagnoses [32, 33] and mixed populations including CRCS [5, 8]. Pain, bloating, tension, and heaviness in the abdomen are symptoms associated with colorectal cancer [34]. These specific symptoms may have initially prompted the diagnosis of colorectal cancer. Consequently, colorectal cancer-specific symptoms evidently act as triggers for FCR and may continue to act as persistent reminders of the disease even after a recurrence has been ruled out.

In this population of long-term CRCS, colorectal cancer-specific symptoms are more strongly correlated to FCR than non-colorectal cancer-specific symptoms. This might indicate that hyper-vigilance, a key characteristic of clinical FCR [11], may be more closely tied to cancer-specific symptoms rather than symptoms in general. Addressing cancer-specific symptom control could potentially mitigate FCR severity, particularly as severe symptoms more effectively differentiate between cFCR and nFCR compared to sFCR and nFCR. However, as this is a cross-sectional study, we cannot make conclusions about causality. Interestingly, no difference in symptom prevalence was observed between CRCS interested in FCR-related support and those who were not.

The mean FCR severity and prevalence of sFCR and cFCR in this study are notably lower than those reported in a recent review [2] and other populations [7, 8]. In the review, which included 676 CRCS, the prevalence rates for nFCR was 50%, sFCR was 35% and cFCR was 16%. However, key differences exist between the populations studied, particularly in terms of age and time since diagnosis. Only 12% of participants in the review cohort were aged 75 or above, whereas this approximates the mean age of participants in the current study. Given that higher FCR is weakly associated with younger age [26], this may partially explain the observed discrepancy. Although time since diagnosis has not been directly linked to FCR, closer proximity to cancer care follow-up visits was associated with higher FCR in the present study. In contrast to the review, where most participants were within five years of diagnosis and likely still undergoing follow-up, most participants in this study had ended cancer care follow-up. Finally, although previous Danish research on CRCS reports a clinical FCR prevalence of approximately 14% [15], differences may reflect variations in national colorectal cancer follow-up guidelines. In Denmark, it is recommended that FCR should be acknowledged and addressed. However, the current Danish standard for assessing psychological distress, the Distress Thermometer, has been shown to inadequately capture FCR [35]. Existing FCR guidelines [36] recommend screening for FCR using the FCR-1r [37], which has been translated and validated in Danish [38].

Three-quarters of survivors experiencing cFCR expressed an interest in engaging in treatment, indicating that the substantial unmet need for psychological interventions targeted at managing FCR also exists among long-term survivors. This highlights the critical importance of integrating tailored FCR management strategies into routine survivorship care to prevent the progression of persistent FCR. The clustering psychological symptoms, including suicidal risk, further underscores the need of comprehensive clinical assessments to identify and prioritize treatment for the most impactful psychological issue as a first step.

The finding that men were more likely to be in the group interested in FCR treatment was surprising [12], although it was reported before [39]. It might be of importance that the survey using the FCRI-SF was sent pro-actively and automatically to all Danish CRCS, with the level of FCR assessed quickly and an option to be directly contacted about participating in a trial on FCR treatment. This straightforward and anonymous approach might particularly appeal to men.

### Limitations

This study reports data from a large, population-based sample size with linkage to register data. While a 56.4% response rate is expected and accepted for PROM studies, it introduces risk of selection bias in estimates of PROM outcomes. High levels of FCR may be associated with either overuse or underuse of healthcare services, which could affect the selection of responders if the survey is perceived as healthcare related, thereby introducing over- or underestimation of FCR prevalence. However, previous research from this cohort has suggested that participation was not associated with demographic and cancer related characteristics, and presumable not FCR [40], suggesting generalizability within the colorectal cancer population. Nonetheless, the study’s findings may be limited by the homogeneity of the CRCS sample, which could affect generalizability to other cancer populations.

Additionally, in this study missing data in the PROMs might lead to an underestimation of symptom presence and severity, as missing values are interpreted as indicating “no symptoms” or a score of zero. Sensitive items, such as those addressing sexual dysfunction, may contribute to missing data, potentially underestimating the prevalence of such late effects in the population. The self-reported nature of PROMs also introduces the potential for social desirability bias.

The FCRI-SF may not be well-suited for assessing FCR levels in long-term survivors, as one item is time-related and assigns 4 points if the responder has been worried about recurrence for several years. This could lead to an overestimation of FCR prevalence among long-term cancer survivors. Further research is needed to evaluate the scale’s ability to accurately discriminate within this population. Similarly, no established cutoffs have been published for the Whiteley-6-R yet. Finally, all “cases” in this study are based on PROMs, which could cause an overestimation of cases. Ideally, cases should have been confirmed by a diagnostic interview.

Respondents with sFCR were not evaluated further; however, this population might also be eligible for treatment. Data on treatment preferences were not collected. Finally, the cross-sectional nature of this study limits our ability to examine causality, and the results may therefore be interpreted with this caution in mind.

## Conclusion

FCR is a common concern among long-term CRCS and severity of FCR is strongly associated with specific colorectal symptoms, tiredness, and psychological symptoms. Providing support for FCR as part of standard hospital-based follow-up may be beneficial, and addressing cancer-specific physical symptoms could be a promising strategy for reducing FCR. Further research is needed to explore this approach.

## Supplementary Information

Below is the link to the electronic supplementary material.

Supplementary file1 (PDF 343 KB)

## Data Availability

Data is provided within the manuscript or supplementary information files.
