# Supplementary material for: Fear of cancer recurrence in long-term colorectal cancer survivors: a nationwide cross-sectional study
Source: J Cancer Surviv. 2025 Jan 27;20(4):1431–42. doi: 10.1007/s11764-025-01746-z (PMC13375672; doi:10.1007/s11764-025-01746-z)
Supplement: Supplementary file 1 — Supplementary file1 (PDF 343 KB) [file 11764_2025_1746_MOESM1_ESM.pdf]

Supplementary File 1.

Article Title: Fear of cancer recurrence in long-term colorectal cancer survivors - A nationwide cross-sectional study

Journal name: Journal of Cancer Survivorship

Author names: Johanne Dam Lyhne, Lars Henrik Jensen, Per Fink, Signe Timm, Lisbeth Frostholm, Allan 'Ben' Smith

Affiliation and email of corresponding author: Department of Oncology, University Hospital of Southern Denmark, Vejle, Denmark. Johanne.Dam.Lyhne@rsyd.dk

Fig. 2. *DAG model visualizing choice of confounders for anxiety/depression as an example*

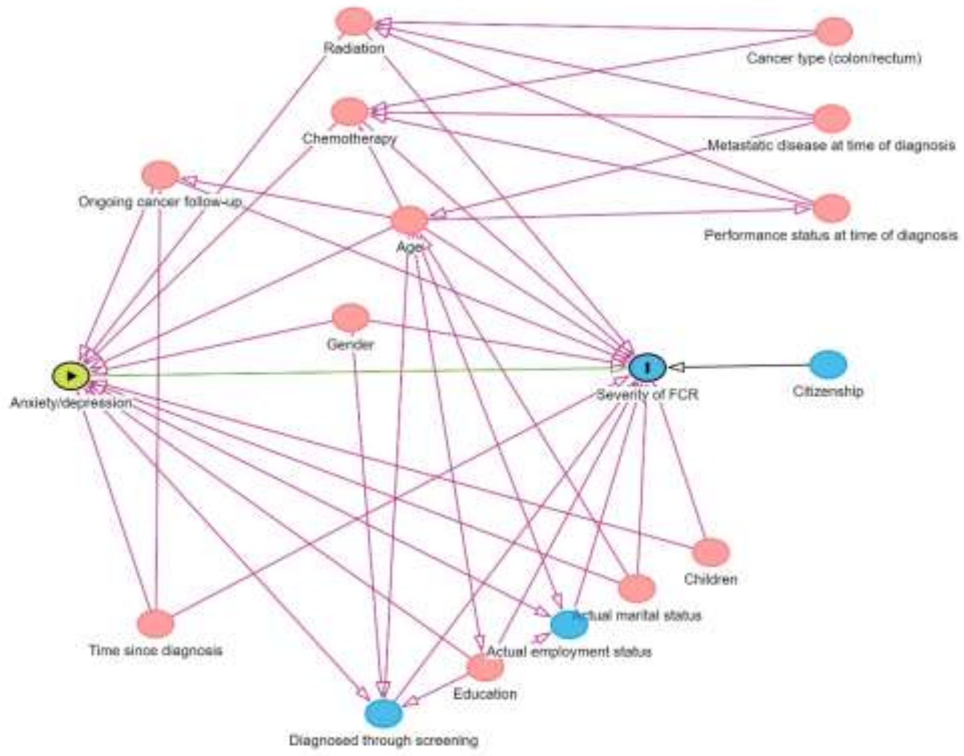

Supplementary File 2

Article Title: Fear of cancer recurrence in long-term colorectal cancer survivors - A nationwide cross-sectional study

Journal name: Journal of Cancer Survivorship

Author names: Johanne Dam Lyhne, Lars Henrik Jensen, Per Fink, Signe Timm, Lisbeth Frostholm, Allan 'Ben' Smith

Affiliation and email of corresponding author: Department of Oncology, University Hospital of Southern Denmark, Vejle, Denmark. Johanne.Dam.Lyhne@rsyd.dk

Table 2a. EQ-5D-5L health states and associations with the severity levels of fear of cancer recurrence.

FCR severity level	Total<br><br>n = 5,435	Minimal or mild FCR (0-13)<br>n = 3,592	Subclinical FCR (13-21)<br>n = 1,555	Clinical FCR (22-36)<br>n = 288
Performance status (EQ-5D-5L)				
Mobility				
1	4,052 (74.5%)	2,767 (77.0%)	1,115 (71.7%)	170 (59.0%)
2	759 (14.0%)	460 (12.8%)	250 (16.1%)	49 (17.0%)
3	404 (7.4%)	240 (6.7%)	124 (8.0%)	40 (13.9%)
4	197 (3.6%)	109 (3.0%)	60 (3.9%)	28 (9.7%)
5	23 (0.4%)	16 (0.5%)	6 (0.4%)	1 (0.4%)
Self-care				
1	5,059 (93.1%)	3,395 (94.5%)	1,431 (92.0%)	233 (80.9%)
2	258 (4.8%)	134 (3.7%)	92 (5.9%)	32 (11.1%)
3	79 (1.5%)	40 (1.1%)	21 (1.4%)	18 (6.3%)
4	24 (0.4%)	12 (0.3%)	8 (0.5%)	4 (1.4%)
5	15 (0.3%)	11 (0.3%)	3 (0.2%)	1 (0.4%)
Activity				
1	3,863 (71.1%)	2,738 (76.2%)	987 (63.5%)	138 (47.9%)
2	933 (17.2%)	522 (14.5%)	350 (22.5%)	61 (21.2%)
3	424 (7.8%)	225 (6.3%)	153 (9.8%)	46 (16.0%)
4	157 (2.9%)	67 (1.9%)	54 (3.5%)	36 (12.5%)
5	58 (1.1%)	40 (1.1%)	11 (0.7%)	7 (2.4%)
Pain				
1	3,331 (61.3%)	2,507 (69.8%)	733 (47.1%)	91 (31.6%)
2	1,373 (25.3%)	757 (21.1%)	526 (33.8%)	90 (31.3%)
3	595 (11.0%)	273 (7.6%)	242 (15.6%)	80 (27.8%)
4	125 (2.3%)	52 (1.5%)	49 (3.2%)	24 (8.3%)
5	11 (0.2%)	3 (0.1%)	5 (0.3%)	3 (1.0%)
Anxiety				
1				
2	4,279 (78.7%)	3,229 (89.9%)	964 (62.0%)	86 (29.9%)
3	863 (15.9%)	298 (8.3%)	451 (29.0%)	114 (39.6%)
4	244 (4.5%)	57 (1.6%)	123 (7.9%)	64 (22.2%)
5	42 (0.8%)	8 (0.2%)	11 (0.7%)	23 (8.0%)
	7 (0.1%)	0	6 (0.4%)	1 (0.4%)

Table 2b. Patient-reported psychological symptoms and associations with the severity levels of fear of cancer recurrence.

FCR severity level	Total<br><br>n = 5,435	Minimal or mild FCR (0-13)<br>n = 3,592	Subclinical FCR (13-21)<br>n = 1,555	Clinical FCR (22-36)<br>n = 288
Anxiety, mean value (sd)<br>(Scale from 0-16)<br>(Cut-off ≥ 6)	1.1 (2.0)	0.6 (1.3)	1.8 (2.3)	4.1 (3.4)
No anxiety (0-5)	5,161 (95.0%)	3,528 (98.2%)	1,428 (91.8%)	205 (71.2%)
Anxiety (6-16)	247 (4.5%)	46 (1.3%)	118 (7.6%)	83 (28.8%)
Missing	27 (0.5%)	18 (0.5%)	9 (0.6%)	0
Depression, mean value (sd)<br>(Scale from 0-24)<br>(Cut-off ≥ 9)	1.3 (2.6)	0.7 (1.7)	2.1 (3.1)	4.5 (4.8)
No depression (0-8)	5,246 (96.5%)	3,548 (98.8%)	1,462 (94.0%)	236 (81.9%)
Depression (9-24)	160 (2.9%)	26 (0.7%)	83 (5.3%)	51 (17.7%)
Missing	29 (0.5%)	18 (0.5%)	10 (0.6%)	1 (0.4%)
Health anxiety, mean value (sd)<br>(scale from 0-24)<br>(Cut-off ≥ 6)	2.6 (3.7)	1.4 (2.6)	4.2 (3.8)	9.0 (5.5)
No health anxiety (0-5)	4,542 (83.6%)	3,333 (92.8%)	1,120 (72.0%)	89 (30.9%)
Health anxiety (6-24)	867 (16.0%)	240 (6.7%)	428 (27.5%)	199 (69.1%)
Missing	26 (0.5%)	19 (0.5%)	7 (0.5%)	0

Table 2c. Patient-reported colorectal cancer specific symptoms and associations with the severity levels of fear of cancer recurrence.

FCR severity level	Total<br><br>n = 5,435	Minimal or mild FCR (0-13)<br>n = 3,592	Subclinical FCR (13-21)<br>n = 1,555	Clinical FCR (22-36)<br>n = 288
Frequent, loose stools (%)				
No problem (0)	2,299 (42.3%)	1,761 (49.0%)	474 (30.5%)	64 (22.2%)
Mild problem (1-2)	2,347 (43.2%)	1,465 (40.8%)	756 (48.6%)	126 (43.8%)

Severe problem (3-4)	729 (13.4%)	327 (9.1%)	305 (19.6%)	97 (33.7%)
Missing	60 (1.1%)	39 (1.1%)	20 (1.3%)	1 (0.4%)
Abdominal pain (%)				
No problem (0)	3,597 (66.2%)	2,734 (76.1%)	776 (50.0%)	87 (30.2%)
Mild problem (1-2)	1,530 (28.2%)	722 (20.1%)	660 (42.4%)	148 (51.4%)
Severe problem (3-4)	180 (3.3%)	48 (1.3%)	85 (5.5%)	47 (16.3%)
Missing	128 (2.4%)	88 (2.5%)	34 (2.2%)	6 (2.1%)
Abdominal bloating, tension, or heaviness (%)				
No problem (0)	2,992 (55.1%)	2,343 (65.2%)	585 (37.6%)	64 (22.2%)
Mild problem (1-2)	2,019 (37.2%)	1,093 (30.4%)	787 (50.6%)	139 (48.3%)
Severe problem (3-4)	350 (6.4%)	104 (2.9%)	162 (10.4%)	84 (29.2%)
Missing	74 (1.4%)	52 (1.5%)	21 (1.4%)	1 (0.4%)
Diarrhea (%)				
No problem (0)	3,462 (63.7%)	2,527 (70.4%)	824 (53.0%)	111 (38.5%)
Mild problem (1-2)	1,540 (28.3%)	869 (24.2%)	563 (36.2%)	108 (37.5%)
Severe problem (3-4)	362 (6.7%)	150 (4.2%)	146 (9.4%)	66 (22.9%)
Missing	71 (1.3%)	46 (1.3%)	22 (1.4%)	3 (1.0%)
Involuntary passage of gas/loose stool (%)				
No problem (0)	2,813 (51.8%)	2,086 (58.1%)	627 (40.3%)	100 (34.7%)
Mild problem (1-2)	2,015 (37.1%)	1,232 (34.3%)	672 (43.2%)	111 (38.5%)
Severe problem (3-4)	518 (9.5%)	222 (6.2%)	224 (14.4%)	72 (25.0%)
Missing	89 (1.6%)	52 (1.5%)	32 (2.1%)	5 (1.7%)
Difficulty emptying bowels during toilet visits (%)				
No problem (0)	2,864 (52.7%)	2,148 (59.8%)	625 (40.2%)	91 (31.6%)
Mild problem (1-2)	1,969 (36.2%)	1,190 (33.1%)	657 (42.3%)	122 (42.4%)
Severe problem (3-4)	508 (9.4%)	199 (5.5%)	240 (15.4%)	69 (24.0%)
Missing	94 (1.7%)	55 (1.5%)	33 (2.1%)	6 (2.1%)
Defecation urgency (%)				
No problem (0)	2,669 (49.1%)	2,032 (56.6%)	552 (35.5%)	85 (29.5%)
Mild problem (1-2)	2,035 (37.4%)	1,235 (34.4%)	682 (43.9%)	118 (41.0%)
Severe problem (3-4)	640 (11.8%)	273 (7.6%)	286 (18.4%)	81 (28.1%)
Missing	91 (1.7%)	52 (1.5%)	35 (2.3%)	4 (1.4%)
Involuntary urination (%)				
No problem (0)	3,863 (71.1%)	2,659 (74.0%)	1,031 (66.3%)	173 (60.1%)
Mild problem (1-2)	1,331 (24.5%)	806 (22.4%)	440 (28.3%)	85 (29.5%)
Severe problem (3-4)	196 (3.6%)	93 (2.6%)	74 (4.8%)	29 (10.1%)
Missing	45 (0.8%)	34 (1.0%)	10 (0.6%)	1 (0.4%)
Frequent urination (%)				
No problem (0)	2,669 (49.1%)	1,876 (52.2%)	674 (43.3%)	119 (41.3%)
Mild problem (1-2)	2,217 (40.8%)	1,429 (39.8%)	683 (43.9%)	105 (36.5%)

Severe problem (3-4)<br>Missing	503 (9.3%)<br>46 (0.9%)	257 (7.2%)<br>30 (0.8%)	183 (11.8%)<br>15 (1.0%)	63 (21.9%)<br>1 (0.4%)
Difficulty emptying bladder (%)				
No problem (0)<br>Mild problem (1-2)<br>Severe problem (3-4)<br>Missing	3,648 (67.1%)<br>1,451 (26.7%)<br>276 (5.1%)<br>60 (1.1%)	2,518 (70.1%)<br>890 (24.8%)<br>147 (4.1%)<br>37 (1.0%)	960 (61.7%)<br>484 (31.1%)<br>88 (5.7%)<br>23 (1.5%)	170 (59.0%)<br>77 (26.7%)<br>41 (14.2%)<br>0
Dry mouth (%)				
No problem (0)<br>Mild problem (1-2)<br>Severe problem (3-4)<br>Missing	3,404 (62.6%)<br>1,582 (29.1%)<br>362 (6.7%)<br>87 (1.6%)	2,430 (67.7%)<br>965 (26.9%)<br>140 (3.9%)<br>57 (1.6%)	848 (54.5%)<br>527 (33.9%)<br>154 (9.9%)<br>26 (1.7%)	126 (43.8%)<br>90 (31.3%)<br>68 (23.6%)<br>4 (1.4%)
Sexual function (%)				
No problem (0)<br>Mild problem (1-2)<br>Severe problem (3-4)<br>Missing	3,109 (57.2%)<br>1,220 (22.5%)<br>954 (17.6%)<br>152 (2.8%)	2,166 (60.3%)<br>770 (21.4%)<br>542 (15.1%)<br>114 (3.2%)	811 (52.2%)<br>387 (24.9%)<br>322 (20.7%)<br>35 (2.3%)	132 (45.8%)<br>63 (21.9%)<br>90 (31.3%)<br>3 (1.0%)
Unpleasant numbness or sensory disturbances (%) (only CRCS who received chemotherapy)				
No problem (0)<br>Mild problem (1-2)<br>Severe problem (3-4)<br>Missing	1,348 (64.5%)<br>532 (25.5%)<br>182 (8.7%)<br>27 (1.3%)	898 (71.7%)<br>264 (21.1%)<br>72 (5.8%)<br>18 (1.4%)	388 (55.8%)<br>225 (32.3%)<br>77 (11.1%)<br>6 (0.9%)	62 (44.0%)<br>43 (30.5%)<br>33 (23.4%)<br>3 (2.1%)
Tiredness (%)				
No problem (0)<br>Mild problem(1-2)<br>Severe problem (3-4)<br>Missing	1,806 (33.2%)<br>2,977 (54.8%)<br>606 (11.2%)<br>46 (0.9%)	1,461 (40.7%)<br>1,863 (51.9%)<br>240 (6.7%)<br>28 (0.8%)	316 (20.3%)<br>972 (62.5%)<br>250 (16.1%)<br>17 (1.1%)	29 (10.1 %)<br>142 (49.3%)<br>116 (40.3%)<br>1 (0.4%)
Total symptom load from the BDS checklist (scale score 0-100) (no established cut-off exists)				
Below the median of 10<br>Above the median of 10<br>Missing	2,660 (48.9%)<br>2,749 (50.6%)<br>26 (0.5%)	2,147 (59.8%)<br>1,431 (39.8%)<br>14 (0.4%)	487 (31.3%)<br>1,057 (68.0%)<br>11 (0.7%)	26 (9.0%)<br>261 (90.6%)<br>1 (0.4%)
Non-gastrointestinal symptom load displayed as a summary of non-gastrointestinal symptoms from the BDS checklist (scale score 0-72)				

(no established cut-off exists)				
Below the median (< 7)	2,590 (47.7%)	2,047 (57.0%)	509 (32.7%)	34 (11.8%)
Above the median (≥ 7)	2,831 (52.1%)	1,534 (42.7%)	1,043 (67.1%)	254 (88.2%)
Missing	14 (0.3%)	11 (0.3%)	3 (0.2%)	0

Supplementary File 3.

Article Title: Fear of cancer recurrence in long-term colorectal cancer survivors - A nationwide cross-sectional study

Journal name: Journal of Cancer Survivorship

Author names: Johanne Dam Lyhne, Lars Henrik Jensen, Per Fink, Signe Timm, Lisbeth Frostholm, Allan 'Ben' Smith

Affiliation and email of corresponding author: Department of Oncology, University Hospital of Southern Denmark, Vejle, Denmark. Johanne.Dam.Lyhne@rsyd.dk

Table 3a. *Associations between physical and psychological symptoms and the interest in engaging in treatment for fear of cancer recurrence.*

	Interested in treatment	Not interested in treatment
Anxiety, mean value (sd)<br>(Scale from 0-16)<br>(Cut-off 6)		
No anxiety symptoms (0-5)	148 (68.8%)	57 (78.1%)
Anxiety symptoms (6-16)	67 (31.2%)	16 (21.9%)
Missing	0	0
Depression, mean value (sd)<br>(Scale from 0-24)<br>(Cut-off 9)		
No depression (0-8)	175 (81.4%)	61 (83.6%)
Depression symptoms (9-24)	39 (18.1%)	12 (16.4%)
Missing	1 (0.5%)	0
Suicidal risk, n (%)<br>(scale from 0-4)		
No problem (0)	197 (91.6%)	68 (93.2%)
Mild problem (1-2)	11 (5.1%)	2 (2.7%)
Severe problem (3-4)	6 (2.8%)	3 (4.1%)
Missing	1 (0.5%)	0
Frequent, loose stools		
No problem (0)	49 (22.8%)	15 (20.6%)
Mild problem (1-2)	91 (42.3%)	35 (48.0%)
Severe problem (3-4)	74 (34.4%)	23 (31.5%)
Missing	1 (0.5%)	0
Abdominal pain		
No problem (0)	62 (28.8%)	25 (34.3%)
Mild problem (1-2)	111 (51.6%)	37 (50.7%)
Severe problem (3-4)	38 (17.7%)	9 (12.3%)
Missing	4 (1.9%)	2 (2.7%)

Abdominal bloating, tension, or heaviness		
No problem (0)	48 (22.3%)	16 (21.9%)
Mild problem (1-2)	106 (49.3%)	33 (45.2%)
Severe problem (3-4)	60 (27.9%)	24 (32.9%)
Missing	1 (0.5%)	0
Diarrhea		
No problem (0)	81 (37.7%)	30 (41.1%)
Mild problem (1-2)	79 (36.7%)	29 (39.7%)
Severe problem (3-4)	52 (24.2%)	14 (19.2%)
Missing	3 (1.4%)	0
Involuntary passage of gas/loose stool		
No problem (0)		
Mild problem (1-2)	73 (34.0%)	37 (37.0%)
Severe problem (3-4)	80 (37.2%)	31 (42.5%)
Not reported	59 (27.4%)	13 (17.8%)
	3 (1.4%)	2 (2.7%)
Difficulty emptying bowels during toilet visits		
No problem (0)	67 (31.2%)	24 (32.9%)
Mild problem (1-2)	92 (42.8%)	30 (41.1%)
Severe problem (3-4)	50 (23.3%)	19 (26.0%)
Missing	6 (2.8%)	0
Defecation urgency		
No problem (0)	60 (27.9%)	25 (34.3%)
Mild problem (1-2)	88 (40.9%)	30 (41.1%)
Severe problem (3-4)	63 (29.3%)	18 (24.7%)
Missing	4 (1.9%)	0
Involuntary urination		
No problem (0)	128 (59.5%)	45 (61.6%)
Mild problem (1-2)	66 (30.7%)	19 (26.0%)
Severe problem (3-4)	20 (9.3%)	9 (12.3%)
Missing	1 (0.5%)	0
Frequent urination		
No problem (0)	80 (37.2%)	39 (53.4%)
Mild problem (1-2)	87 (40.5%)	18 (24.7%)
Severe problem (3-4)	47 (21.9%)	16 (21.9%)
Missing	1 (0.5%)	0
Difficulty emptying bladder		
No problem (0)	125 (58.1%)	45 (61.6%)
Mild problem (1-2)	56 (26.1%)	21 (28.8%)
Severe problem (3-4)	34 (15.8%)	7 (9.6%)
Missing	0	0
Sexual dysfunction		
No problem (0)	94 (43.7%)	38 (52.1%)
Mild problem (1-2)	50 (23.3%)	13 (17.8%)
Severe problem (3-4)	69 (32.1%)	21 (28.8%)
Missing	2 (0.9%)	1 (1.4%)
Neuropathy		
No problem (0)	105 (48.8%)	45 (61.6%)
Mild problem (1-2)	68 (31.6%)	19 (26.0%)

Severe problem (3-4)	39 (18.1%)	8 (11.0%)
Missing	3 (1.4%)	1 (1.4%)
Tiredness		
No problem (0)	20 (9.3%)	9 (12.3%)
Mild problem (1-2)	105 (48.8%)	37 (50.7%)
Severe problem (3-4)	89 (41.4%)	27 (37.0%)
Missing	1 (0.5%)	0
